# Probiotic Bacteria Induce a ‘Glow of Health’

**DOI:** 10.1371/journal.pone.0053867

**Published:** 2013-01-16

**Authors:** Tatiana Levkovich, Theofilos Poutahidis, Christopher Smillie, Bernard J. Varian, Yassin M. Ibrahim, Jessica R. Lakritz, Eric J. Alm, Susan E. Erdman

**Affiliations:** 1 Division of Comparative Medicine, Massachusetts Institute of Technology, Cambridge, Massachusetts, United States of America; 2 Laboratory of Pathology, Faculty of Veterinary Medicine, Aristotle University of Thessaloniki, Thessaloniki, Greece; 3 Civil and Environmental Engineering, Massachusetts Institute of Technology, Cambridge, Massachusetts, United States of America; 4 Biological Engineering, Massachusetts Institute of Technology, Cambridge Massachusetts, United States of America; 5 Broad Institute of MIT and Harvard, Cambridge, Massachusetts, United States of America; Public Health Agency of Canada, Canada

## Abstract

Radiant skin and hair are universally recognized as indications of good health. However, this ‘glow of health’ display remains poorly understood. We found that feeding of probiotic bacteria to aged mice induced integumentary changes mimicking peak health and reproductive fitness characteristic of much younger animals. Eating probiotic yogurt triggered epithelial follicular anagen-phase shift with sebocytogenesis resulting in thick lustrous fur due to a bacteria-triggered interleukin-10-dependent mechanism. Aged male animals eating probiotics exhibited increased subcuticular folliculogenesis, when compared with matched controls, yielding luxuriant fur only in probiotic-fed subjects. Female animals displayed probiotic-induced hyperacidity coinciding with shinier hair, a feature that also aligns with fertility in human females. Together these data provide insights into mammalian evolution and novel strategies for integumentary health.

## Introduction

Skin and mucosal surfaces of mammalian species are populated by millions of bacteria that impart diverse metabolic effects [1]. These host-associated microbes play a well-established role in homeostasis in the gastrointestinal (GI) tract [2], [3]. There is now substantial evidence linking various gut microbiota and local immunity networks with systematic effects on the immune system [4], [5], [6]. Disruption of the normal balance between microbial communities in the intestine is associated with allergic, autoimmune, metabolic, and neoplastic pathologies in the GI tract and other distant tissues [7], [8], [9], [10], [11]. Along these lines, experimental and clinical studies have shown that the dietary enrichment with certain ‘probiotic’ organisms activates immune and metabolic pathways that restore tissue homeostasis and promote overall health [12], [13], [14].

An exaggerated paradigm of an organ distal from the bowel that could benefit from probiotic consumption is the skin. Interestingly, research data from both mice and humans suggest that dietary supplementation with probiotic lactic acid bacteria has beneficial effects in the skin [14], [15], [16], [17], [18]. The importance of probiotic effects on skin extends beyond obvious cosmetic aspects to broader host health. Indeed, the appearance of the skin and its appendages has been considered by medicine traditions worldwide as a clinical sign of good health.

In a previous GI immune-related study, we documented changes in fur appearance in mice treated with probiotics. Similar ‘probiotic’ organisms dominate under natural conditions during infancy and fertility in many animal species [7], [8], [9]. Taken together these facts lead us to hypothesize that probiotics may play a role in effecting the ‘glow of health’ associated with youth and reproductive fitness. Further, we postulated that feeding of probiotic organisms recapitulates these beneficial integumentary effects characteristic of youth within aged adult animals.

## Results

### Mice have Shinier Fur After Eating Probiotic Yogurt

**Figure 1:**
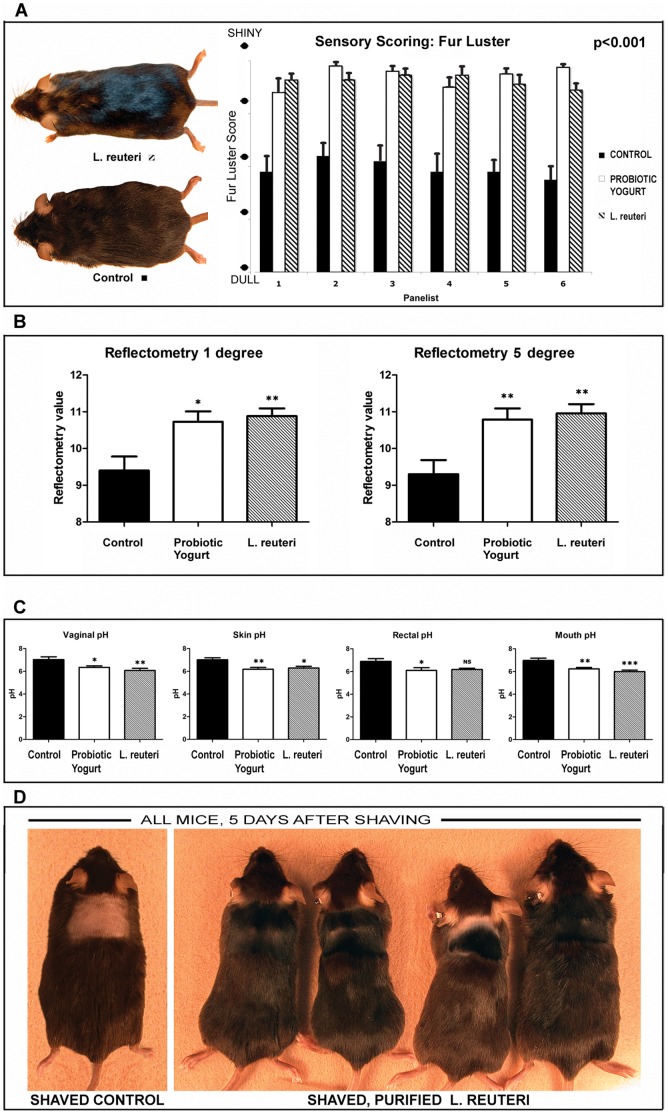
Dietary supplementation with probiotic organisms induces lustrous fur in aged mice. (**A**) Aged female C57BL/6 strain mice consuming probiotics exhibited significantly (p<0.0001) increased shininess quantifiable by sensory evaluation of human panelists blinded to mouse identity and using a standardized fur luster scale. (**B**) Reflectometry instrumentation using one degree (p<0.01) and five degree (p<0.01) field analyses revealed significantly (p<0.05) increased light reflectivity of mouse fur after eating probiotics, when tested under highly controlled lighting conditions. (**C**) Evaluation of mucocutaneous pH shows eating probiotic yogurt or purified probiotic bacteria induced more acidic conditions in skin, oral cavity, vaginal mucosa, and rectum of female mice. Similar trends in male animals did not reach statistical significance. (**D**) C57BL/6 mice consuming purified *L. reuteri* bacteria in drinking water had more rapid fur re-growth after shaving (right) when compared with matched mice drinking regular water (left).

We discovered unusually lustrous fur in mouse models while feeding probiotic yogurt during gastrointestinal studies. We subsequently examined these animals to test specific roles for probiotic organisms on integument. Inbred C57BL/6 mice were fed yogurt starting at 20–24 weeks of age and then examined at 40–48 wks of age (20–24 weeks later) using mechanical and sensory methods of fur luster evaluation, tissue pH readings, and histology. Surprisingly, differences in fur luster were observed within as few as seven days after feeding of probiotic yogurt to inbred C57BL/6 mice (Figure 1A). In contrast, age-matched animals receiving control chow alone had dull fur and suffered from occasional alopecia and dermatitis. Differences in light reflectivity between treatment groups were quantifiable by sensory evaluation (p<0.0001) using a standardized luster scale to complement mechanical scoring (Fig. 1A), and then also by commercially available reflectometry using one degree (p<0.01) and five degree field analyses (p<0.01) (Fig. 1B), under highly controlled lighting conditions. These differences in fur luster were highly significant in female animals. However, a similar trend toward probiotic-induced shininess was not statistically significant in males (data not shown).

### Eating Probiotic Yogurt Increases Dermal Thickness

**Figure 2:**
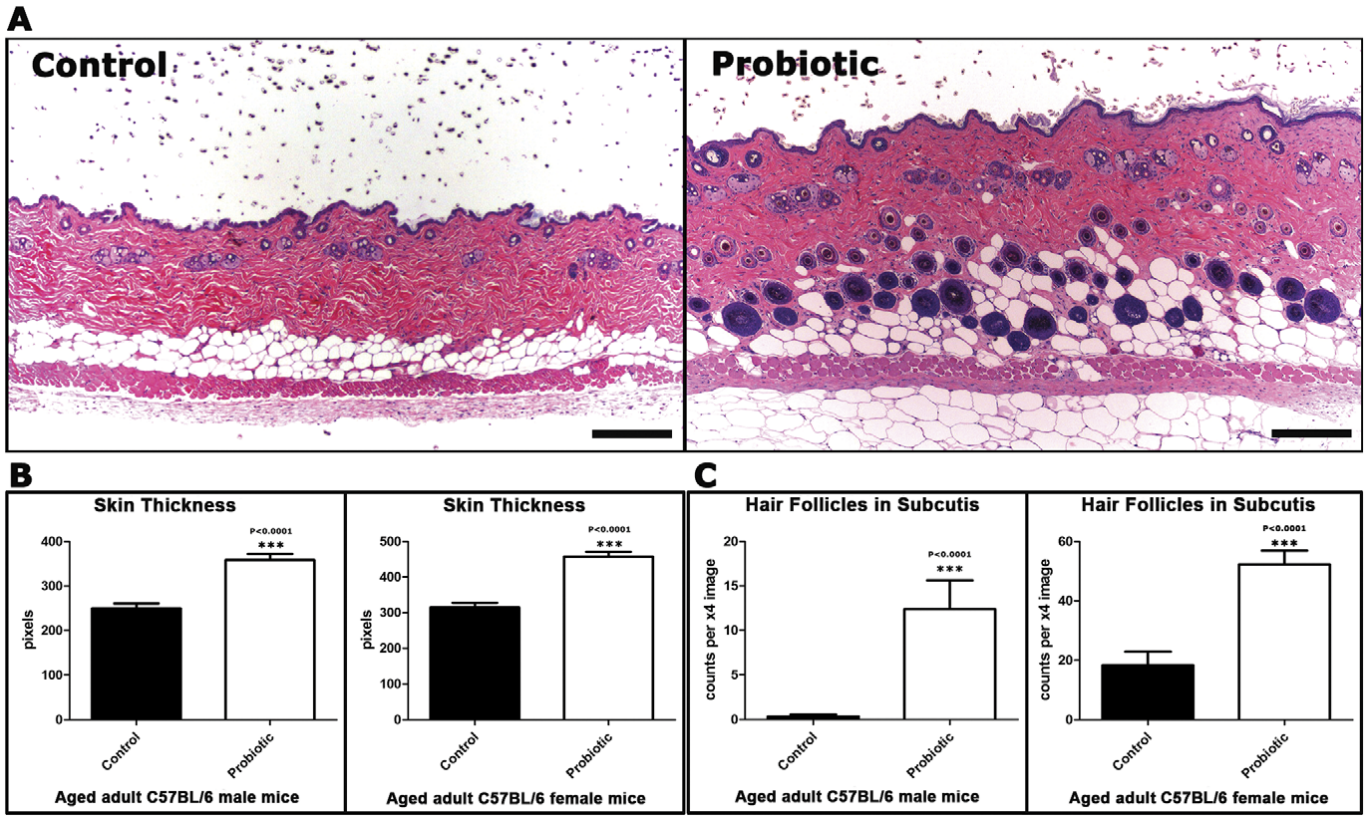
Dietary supplementation with probiotics affects skin histology of mice. (**A**) Probiotic-fed C57BL/6 male mice differ from their control diet-fed counterparts by having subcutaneous hair follicle profiles and a thicker skin. Hematoxylin and Eosin. Bars = 250 µm. Histomorphometrical analysis reveals significant (P<0.0001) probiotic diet-associated increase in (**B**) skin thickness and (**C**) subcutaneous hair follicles in both genders. The y-axis depicts the mean±SEM of histomorphometric counts in each experimental group.

In order to test associations between eating probiotic yogurt and appearance of hair coat, we first used microscopy–assisted histomorphometry from C57BL/6 mice (Fig. 2A). We found an increase in thickness of dermis (Fig. 2B) in mice supplemented with yogurt when compared with mice consuming control chow alone. Quantitative assessment of skin thickness involved measuring the distance from the epidermis to paniculus carnosus. In C57BL/6 mice, females eating probiotic yogurt had a greater dermal thickness (457.1±64.86 pixels) than control females (314.7±60.06) eating regular chow alone (Fig. 2B). Males displayed a similar trend when eating yogurt (357.9±63.87) compared with control male animals (249.8±48.75). Consumption of probiotic-rich diet correlated with a significant increase of skin thickness (P<0.0001) in both genders.

### Dietary Probiotics Up-regulate Subcuticular Hair Follicle Cycling

Given that skin thickens during the anagen phase of the hair cycle, we next sought to determine diet-associated differences in hair cycling. Mice eating probiotic yogurt displayed significantly more subcuticular hair follicles per low power microscopic field than matched control mice (Fig. 2C). According to the well-established histological parameters of hair follicle development [19], [20], [21], [22], follicles residing in the subcutis are in mid- to late anagen (Anagen IIIb–VI) or catagen (Catagen I–VII) stage of the hair growth cycle but not in the early anagen (Anagen I–IIIa) or telogen stages, leading us to further characterize and quantify hair follicle cycling after feeding of yogurt.

### Anagenic Follicular Shift Arises after Consumption of Probiotic Yogurt

**Figure 3:**
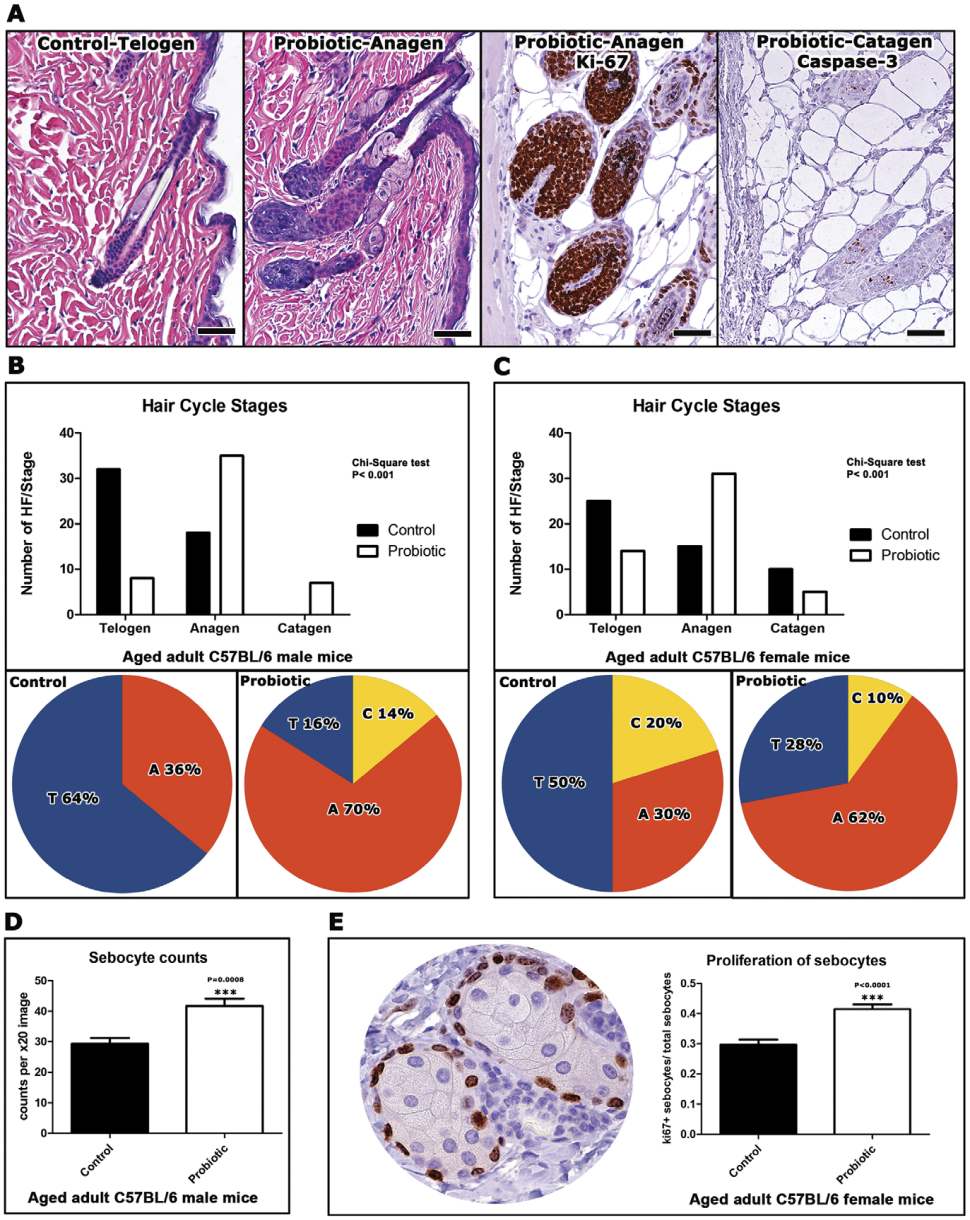
Dietary probiotics increase anagen hair follicles and proliferation of sebocytes in aged mice. (**A**) Quiescent (telogen-phase) hair follicles predominate in control-diet fed C57BL/6 mice. In contrast, the majority of the hair follicles are active (anagen-phase) in probiotic-fed mice of the same age. Active hair follicles undergoing rapid growth exhibit numerous proliferating cells (ki-67+). More rarely the regressing stage (catagen) contain caspase-3+ apoptotic cells. *Control Telogen* and *Probiotic Anagen*: Hematoxylin and Eosin. *Probiotic Anagen-ki-67* and *Probiotic Catagen-caspase-3*: DAB chromogen, Hematoxylin counterstain. Bars = 50 µm. Classification of fifty intact longitudinally-sectioned hair follicles per treatment group were evaluated according to their stage of cycling (in **B** and **C** below). The distribution pattern of hair-follicle staging differs significantly (P<0.0001) among probiotic- and control diet-fed (**B**) male and (**C**) female mice. Numbers on the y-axis of bar graphs represent the mean±SEM of hair-follicles classified in each hair cycle stage. The % percentage of hair follicles in telogen (T), anagen (A) or catagen (C) stage is illustrated in circular graphs. Probiotic-fed mice of both genders show an anagen stage predominance. (**D**) Dietary supplementation with probiotics lead to a significant (P<0.0001) increase of sebocytes in skin pilosebaceous units. The y-axis stands for the mean±SEM of sebocyte counts per X20 high power field image. (**E**) The quantitative assessment of cellular proliferation in sebaceous glands with ki-67-specific immunohistochemistry (circular image) reveals that dietary probiotics increase the proliferative capacity of sebocytes. Numbers on the y axis of bar graphs correspond to the mean±SEM of the index of proliferating sebocytes per total number of sebocytes in x40 high power fields. Circular image: DAB chromogen, Hematoxylin counterstain. Bar = 25 µm.

In order to more accurately probe effects of dietary probiotics in hair follicle cycling, we used longitudinally-oriented hair follicles in histological sections of skin. Hair follicle staging was based on previously published histomorphological criteria together with proliferation and apoptosis-specific immunohistochemistry [19]. We found that probiotic consumption lead to robust hair growth (Figs. 1D and 3A) and significantly different hair staging profiles in mice of both genders (Figs. 3B–C). Mice eating yogurt had more hairs in anagen phase when compared with matched mice fed control diet alone. Specifically, male C57BL/6 mice consuming probiotic yogurt had discrete hairs with characteristics of anagen (70%), catagen (14%) and telogen phase (16%) (Fig. 3B). By contrast, matched mice eating control diet alone exhibited telogen dominance (64%), with less frequent anagen (36%) and no catagen hair follicles. The distribution of hair follicle cycle stages in female C57BL/6 mice was similar with anagen (62%), catagen (10%) and telogen (28%) for yogurt-fed mice, whereas control diet-fed mice had anagen (30%), catagen (20%) and telogen (50%) (Fig. 3C). The yogurt-induced benefit in pellage was evident in aged adult mice with yogurt initiated at age 24 weeks.

### Feeding of Probiotic Yogurt Stimulates Follicular Sebocytes during ‘Glow of Health’

The short time frame associated with the onset of the lustrous phenotype led us to hypothesize rapid involvement of epithelial secretions. We performed morphometric counts of sebocytes to identify histologically any detectable effects of probiotic consumption in sebaceous glands. We found that probiotic yogurt-fed mice had significantly more (P<0.0001) sebocytes per high power field (41.75±10.52) compared with their control diet-fed counterparts (29.35±8.499) (Fig. 3D). To further elaborate on this result we compared the index of ki-67-positive sebocytes per total sebocytes in high power histological fields of the same mice. Again, yogurt-fed mice had a significantly higher (P<0.0001) index of proliferating sebocytes (0.415±0.07) compared to control diet-fed mice (0.2965±0.077) (Fig. 3E). Given that sebum is the product of a holocrine type of secretion, more sebocytes within sebaceous glands translate into more sebum, which in turn may contribute to the shinier appearance of fur induced by the probiotic-enriched diet. Based on these data, we postulated that supplemental feeding of probiotic organisms mimicked naturally- occurring integumentary display during peak health and reproductive fitness.

### Feeding of Probiotics Leads to Acidic pH in Female Mice

It is widely known that an acidic pH alters hair cuticle and contributes to shiny hair. We hypothesized that an acidic pH may help explain the shinier appearance of fur induced by the probiotic-enriched diet in females when compared with male animals. We examined pH levels in C57BL/6 mice consuming probiotic yogurt by sampling their skin, oral cavity, vaginal mucosa, and rectum upon necropsy. We found these tissues were significantly (p<0.001) more acidic in females consuming probiotic yogurt, when compared with animals eating normal mouse chow alone (Fig. 1C). Only the female animals exhibited significantly lower pH after eating probiotic yogurt. A similar trend toward acidic pH and fur sheen was not statistically significant in male mice, although male animals did exhibit sebocytogenesis and especially dense pellage after eating probiotics (Fig. 3B).

### Probiotic Organisms/Acidic pH Coincide with Peak Fertility in Humans

**Figure 4:**
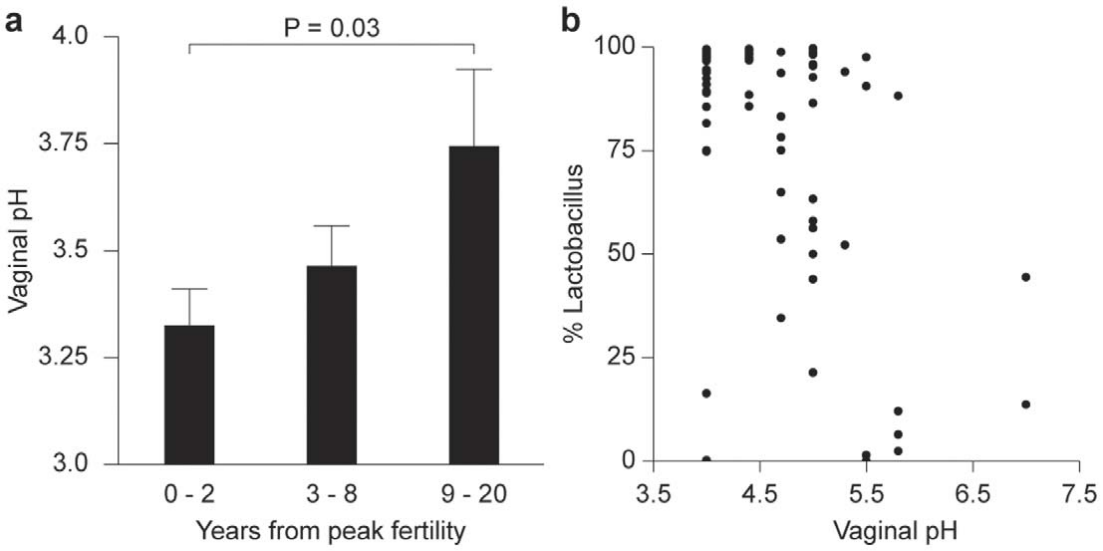
Vaginal pH correlates with Lactobacillus abundance and peak fertility in humans. (**A**) Bars show mean (+SEM) of vaginal pH for women as a function of number of years before or after peak fertility, which was taken to be 25 years of age for all subjects. (**B**) Vaginal pH is significantly associated with *Lactobacillus* abundance (Kendall’s Tau test; τ = −0.38; P = 3.2×10^−8^). Statistical differences between groups were assessed using the Mann-Whitney U test.

We next sought to determine whether probiotic organisms are similarly associated with mucocutaneous pH levels in women. Using published data of Ravel et al (2011) for vaginal microbiome and pH levels of human females [12], we found that an acidic vaginal pH correlated with *Lactobacillus sp* abundance and peak fertility, estimated to be 25 years of age in women (Fig. 4). Taken together with our earlier data, this led us to postulate that probiotic bacteria induce host physiological changes including a more acidic pH resulting in radiant skin and shiny hair signaling peak health and fertility and thus a good reproductive investment.

### Purified Lactobacillus sp Organisms are Sufficient for Lustrous Fur in Mice

**Figure 5:**
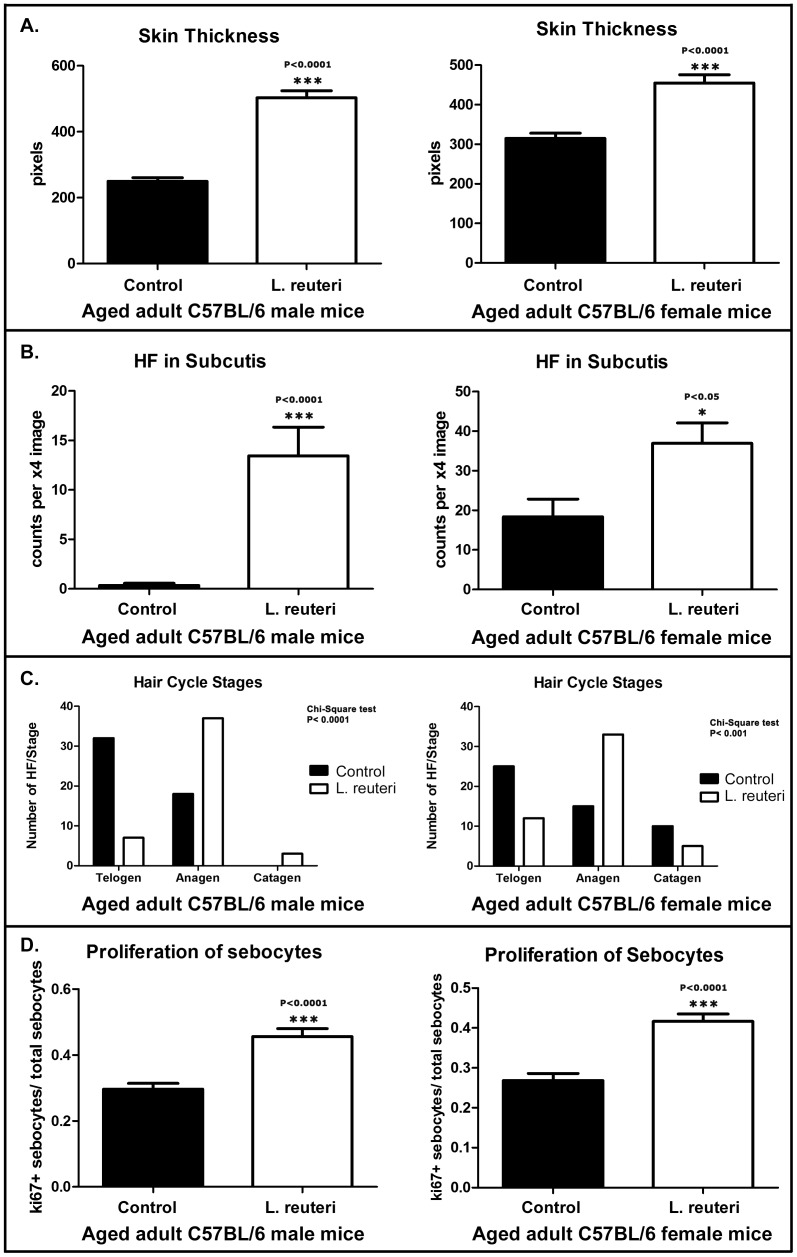
Dietary supplementation with purified *Lactobacillus reuteri* in drinking water mimics effects of eating probiotic yogurt on skin histology of aged mice. Wild type C57BL/6 mice fed purified *L. reuteri* in drinking water differ significantly from their regular water-fed counterparts by having thicker skin and an increased subcutaneous hair follicle and sebocyte profile. (**A**) Histomorphometrical analysis reveals significant (P<0.0001) probiotic diet-associated increase in skin thickness and (**B**) number of subcutaneous hair follicles (P<0.05) in both genders. The y-axis depicts the mean±SEM of histomorphometric counts in each experimental group. (**C**) In mice treated with *L. reuteri* in drinking water, the majority of the hair follicles are active (anagen-phase). In contrast, control mice of the same age have predominantly quiescent (telogen-phase) hair follicles. The distribution pattern of hair-follicle staging differs significantly (P<0.0001) among *L. reuteri*- and control diet-fed in both male and female mice. Numbers on the y-axis of bar graphs represent the mean±SEM of hair follicles classified in each hair cycle stage. (**D**) Further, adding purified *L. reuteri* in drinking water lead to a significant (P<0.0001) increase of sebocytes in skin pilosebaceous units. The y-axis stands for the mean±SEM of sebocyte counts per X20 high power field image. Numbers on the y-axis of bar graphs correspond to the mean±SEM of the index of proliferating sebocytes per total number of sebocytes in x40 high power fields.

In order to test whether it may be the probiotic organisms – instead of the yogurt substrate providing nutrients such as vitamin D [13] – that were responsible for skin health and exuberant hair growth, we fed purified probiotic organisms alone in drinking water to mice, without yogurt supplementation. We found that daily consumption of Lactobacillus reuteri [23], a human microbial isolate proven effective at suppressing colitis, added to drinking water led to lustrous fur similar to that seen in mice eating probiotic yogurt (Figs. 1B–D). This included significantly (p<0.001) thicker skin, (503.1±94.16 vs 249.8±48.75 in males and 455±93.03 vs 314.7±60.06 in females, respectively), more anagen-phase hairs (anagen 74%, catagen 6%, telogen 14% vs anagen 36%, catagen 0% telogen 64% in males and anagen 66%, catagen 10%, telogen 24% vs anagen 30%, catagen 20%, telogen 50% in females), more sebocytes (40.55±7.193 vs 29.35±8,499 in males and 46.05±10.05 vs 25.75±11.72 in females) and a higher sebocyte proliferation index (0.456±0.105 vs 0.296±0.077 in males and 0.416±0.082 vs 0.268±0.08 in females) than mice consuming control chow alone (Figure 5A–D). These data showed that L. reuteri exposure was sufficient for significant physiological changes including more acidic pH levels in mice (Fig. 1D). Furthermore, when directly comparing mice consuming *L. reuteri* in their drinking water *versus* mice eating the probiotic yogurt, there were no significant differences between hair follicles in their subcutis (not significant; p = 0.98 in males and p = 0.06 in females) or in follicular staging (NS; p = 0.44 in males and p = 0.89 in females) or in sebocyte counts (NS; p = 0.89 in males and p = 0.76 in females) and proliferation (NS; p = 0.29 in males and p = 0.76 in females)(Fig. 5) or fur luster (NS; p>0.05) (Fig. 1). Thus, in these mice comparable integumentary benefits were achieved with either consumption of probiotic yogurt or with routine feeding of purified *L. reuteri* organisms.

### Integumentary Health Benefits of Oral Probiotics Require Anti-inflammatory Cytokine Interleukin-10

**Figure 6:**
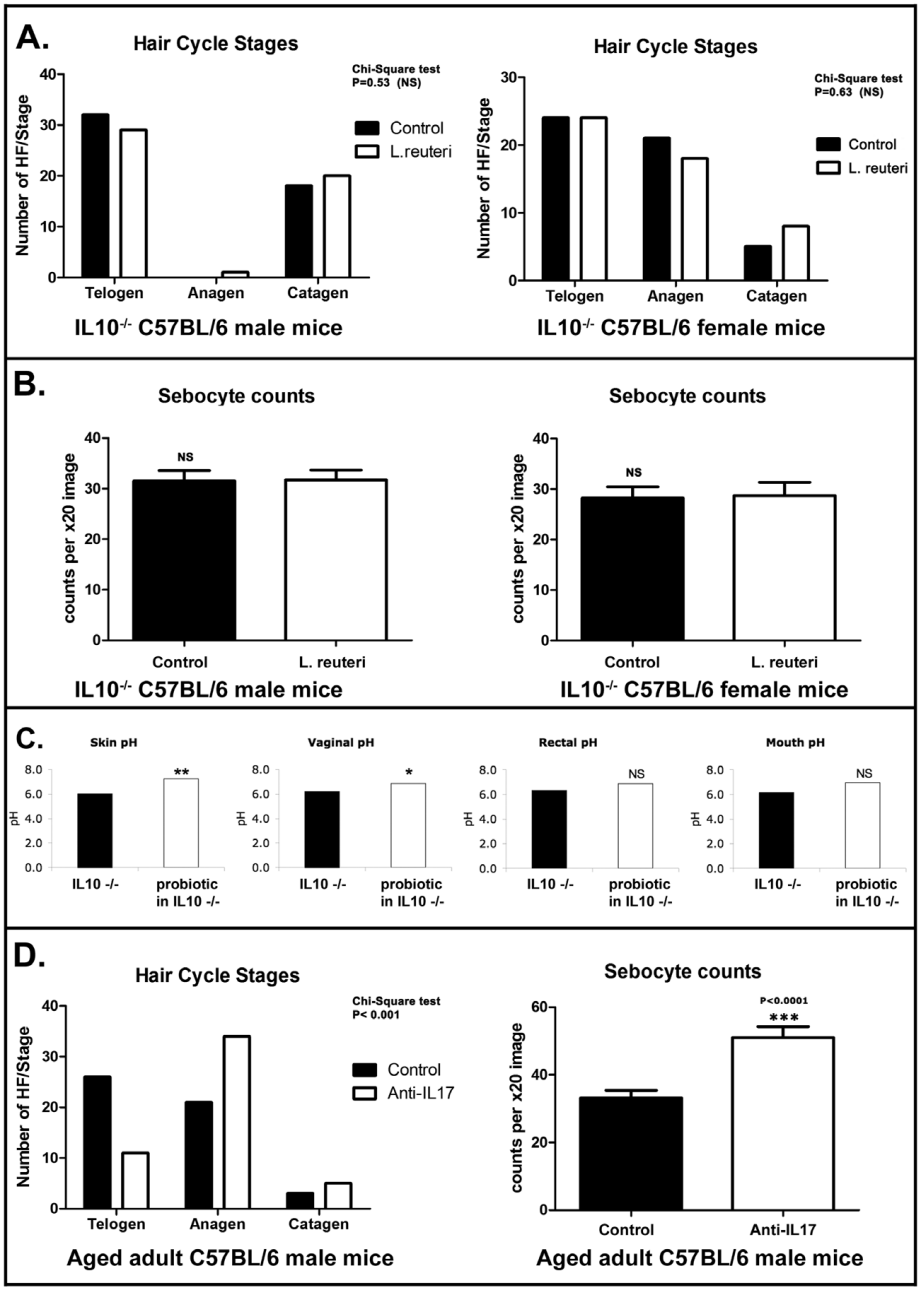
*Lactobacillus reuteri* –induced benefits in hair quality require anti-inflammatory cytokine Interleukin-10. In contrast to wild type animals, feeding of L reuteri to aged C57BL/6 mice lacking interleukin (Il)-10 failed to improve subcutaneous hair follicle or sebocyte profile. (**A**) Histomorphometrical analysis in Il-10-deficient mice reveals insignificant differences in hair follicle activity and distribution (anagen-phase versus telogen-phase). Numbers on the y-axis of bar graphs represent the mean±SEM of hair-follicles classified in each hair cycle stage. (**B**) Likewise, sebocyte counts were not significantly different among *L. reuteri*- and control water-fed Il-10-deficient mice. Numbers on the y-axis of bar graphs represent the mean±SEM of sebocyte counts per X20 high power field image. (**C**) Evaluation of mucocutaneous pH shows eating probiotics induces more alkaline conditions in skin, oral cavity, rectum and vaginal mucosa of Il-10-deficient mice, contrasted with the more acidic conditions in WT female mice (Fig. 1D). (**D**) Depletion of Il-17A using anti-cytokine antibodies recapitulates the probiotic-induced glow of health features in the skin including hair follicle anagen phase predominance and vastly increased numbers of sebocytes in sebaceous glands. Numbers on the y-axis of bar graphs represent the mean±SEM of hair-follicles classified in each hair cycle stage. Numbers on the y-axis of bar graphs represent the mean±SEM of sebocyte counts per X20 high power field image.

We hypothesized that probiotic bacteria imparted integumentary health benefits by an anti-inflammatory mechanism as previously characterized in GI tract mucosa [24] and in skin [25]. To test a possible requirement for anti-inflammatory cytokine Interleukin (Il)-10 in probiotic benefits, we fed *L. reuteri* to C57BL/6 strain mutant mice entirely lacking Il-10. When comparing Il-10-null mice with or without oral *L. reuteri* supplementation, there were no differences in thickness of skin (323.5±99.08 vs 281.9±73.8, p = 0.2184 for males and 244.8±46.93 vs 290.2±131, p = 0.2448 for females), subcutaneous hair follicle profiles (p = 0.6468 for males and p = 0.9235 for females), up-regulation of anagen-phase in hair cycles (p = 0.53 for males and p = 0.63 for females; Fig. 6A), sebocyte counts (31.7±8.874 vs 31.5±9.361, p = 0.9568 for males and 28.7±11.75 vs 28.2±10.09 p = 0.946 for females; Fig. 6B) and sebocyte proliferation index (0.3025±0.07 vs 0.2785±0.08, p = 0.4476 for males and 0.2825±0.07 vs 0.2625±0.07, p = 0.329 for females). Female Il-10-deficient mice exhibited a more alkaline - rather than more acidic - mucocutaneous pH levels when eating probiotics (Fig. 6C). These data demonstrated that the probiotic-triggered features of lustrous fur required Il-10-competency in host animals.

It is a well-established paradigm that Il-10 serves to down-regulate pro-inflammatory cytokines such as Il-17 within bowel and skin [6]. To test whether lowering systemic Il-17 levels may produce similar outcomes to effects of eating probiotic bacteria, we depleted Il-17A in otherwise untreated aged wild type C57BL/6 mice and discovered blocking Il-17 recapitulates the effects of eating probiotics: including significantly increased skin thickness (440.3±76.24 vs 335±145.7, p<0.01), increased hair follicles in subcutis (24.5±26.99 vs 9.1±16.55, p<0.05), hair follicle anagen phase predominance (68% vs 42%) and total sebocytes (51.05±14.51 vs 33.15±10.24, P<0.001) (Fig. 6D), along with increased sebocyte proliferation index (0.427±0.061 vs 0.309±0.079, p<0.001).

Finally, we found that genetically-outbred adult Swiss mice fed purified L. reuteri daily in drinking water exhibited dermal thickening, folliculogenesis, and sebocytogenesis comparable to that displayed above in C57BL/6 wt animals (Fig. 5). Although eating *L. reuteri* apparently imparted sleek pellage in Swiss mice, fur shininess was not specifically quantified in these animals with white fur. Consumption of *L. reuteri* did induce up-regulation of serum protein Il-10 and down-regulation of serum protein Il-17A in Swiss mice (data not shown) mimicking the inflammatory paradigm in C57BL/6 mice. Taken together, these findings demonstrate reproducibility among diverse mouse genetic backgrounds and heightens potential relevancy in other mammalian systems.

## Discussion

Luxuriant hair is widely recognized as a display of health and reproductive fitness. In many mammalian species, healthfulness has recently been attributed to beneficial bacteria [7], [8], [9]. We found that routinely eating probiotic yogurt imparted a healthful glow in animal models, and this effect was reproducible by eating purified *L. reuteri* organisms alone without yogurt supplementation. Although effects imparted by diverse bacterial species classified as ‘probiotics’ may vary widely, we show specifically that *Lactobacillus reuteri* bacteria alone were sufficient for features such as follicular anagen-phase shift, sebocytogenesis, and acidic mucocutaneous pH, and that these events required Il-10, culminating in an epidermal display of fitness. We examined hyperacidity in human subjects finding that *Lactobacillus sp* and acidic pH coincided with life stages of peak fertility, supporting findings of earlier studies in women [12]. We postulate that probiotic-triggered glowing skin and hair typical of youth recreated in our aged animals likely arises from microbe-triggered effects on inflammation within skin. Indeed, mice lacking anti-inflammatory cytokine Il-10 failed to exhibit integumentary benefits after eating probiotics. Inversely, systemic treatment with anti-IL17A antibody mimicked animals feeding on yogurt or *L. reuteri*, supporting an immune-mediated mechanism. This anti-inflammatory effect may emerge systemically from the gastrointestinal (GI) tract [26] impacting both systemic and local immune health [2], [3], [24]. Ingestion of *Lactobacillus sp* was previously shown to dampen stress-related inflammatory responses in the skin through a gut-brain-skin axis [15], at least in part by regulating emotions via the vagus nerve [27]. Alternatively, recent clinical trials indicate topical probiotic lysates may be directly beneficial to skin [28] highlighting the possibility of more local immune-mediated mechanisms of microbes distributed during grooming.

Shiny hair, in particular, is universally described as attractive in women signaling their health and reproductive fitness [29]. Probiotic-induced differences in hair luster in female mice emerged rapidly after feeding yogurt or purified bacteria, leading us to postulate that immediate impact on hair gloss may be due to increased epithelial sebaceous secretions [30]. Sebum is comprised of fatty acids including wax esters [31] that may simultaneously alter pH and fill imperfections in the hair cuticle enhancing reflection of light [32]. For example, lanolin, a waxy sebaceous secretion in sheep, is frequently used in cosmetics to protect human skin and also impart a healthful glow in this way. Increased sebocyte counts after probiotics were seen in mice of both genders; however, significant acidity and shinier fur were seen only in female animals. More frequent grooming activity arising from elevated levels of oxytocin [33] in our mice after feeding probiotics (manuscript in preparation) may help distribute sebum and hasten radiance in probiotic-fed animals. In contrast with *wt* animals, female mice deficient in Il-10 exhibited alkaline skin and mucosae and failed to benefit clinically from probiotic supplementation. This matched prior studies showing Il-10-dependent recruitment of anti-inflammatory immune cells after probiotic consumption [24] and supports the relevancy of microbially-induced inflammation in health. During these studies, phenotypic differences between wild type and Il-10-deficient C57BL/6 animals did not emerge until after feeding of probiotic yogurt or *L. reuteri* (data not shown), leading us to conclude that Il-10 was specifically required for benefit from probiotic bacteria. Precisely how *L. reuteri* and inflammation coincide mechanistically in this process within the pilosebaceous unit remains to be determined. Probiotics may have systematic effects on many inflammatory cells and cytokines, including IL-10, TGF-β1, IL-17, IL-22, IL-1, TNF-α and others [2], [4], [5], [6], [11], which also have been shown to have important roles in skin health and disease [16], [17], [18], [34], [35], and in hair follicle cycling [22], [36].

Hair density has also been associated with peak health and vitality in humans in many cultures [37], [38]. We attributed robust hair growth seen in aged male mice to a follicular shift from predominantly telogen (in control mice) toward a robust 70% of follicles in anagen phase (in probiotic-consuming mice). Both hair growth and increased sebocyte formation are strongly regulated by hormones [39], [40]; in particular, the androgenic hormone testosterone normally causes robust hair growth in young males. Elevated levels of androgen hormones in our male mice after feeding probiotics (manuscript in preparation) may serve to stimulate sebocytes and associated hair follicles in our probiotic-fed animals [40], [41]. During normal aging in humans, telogen effluvium develops as a result of testosterone metabolites such that quiescent telogen phase scalp hairs predominate causing thinning hair [42]. Male pattern baldness is incompletely understood, but is attributed to complex interactions between genetics, hormones and inflammation [43]. Extrapolation from data of mice to humans suggests that excessive inflammation in the form of uncontrolled IL-17A subverts scalp hair growth, and this may be remedied by eating probiotic bacteria such as *L. reuteri*, but interpretation is complicated by disparities in hair on scalp versus other body sites of these species [37]. Nonetheless, aged male mice eating probiotics displayed Il-10-dependent dense fur together with elevated testosterone and increased virility (data not shown) when compared with mice eating control diet alone. It is unknown whether eating of probiotic yogurt may forestall or reverse follicular activities of hormones in human subjects.

Although the skin of both genders of mice improved with dietary probiotics, the female animals showed a more robust response regarding alterations in skin pH and fur luster. This response depended upon the presence of anti-inflammatory cytokine Il-10. In humans, it has been shown that the axillary skin surface pH decreases significantly in women but instead raises in men after simple interventions, such as washing with tap water [44]. The mechanisms underlying such gender related differences in the skin or microbial physiology remain elusive, although they likely reflect the differential effects of sex steroid hormones and associated immunological factors on the skin and mucosal surface secretions found in each gender [45].

We postulate the probiotic-induced ‘glow’ in aged animals not only reflects health but also mimics key aspects of microbial symbiosis enhancing reproductive fitness in mammalian hosts. During periods of fertility, immune and hormonal effects of probiotic organisms dominate environmental interfaces and facilitate host survival and reproductive success. In humans and other mammals, the resulting radiant skin and shiny hair signal a good reproductive investment and are thus inherently attractive to conspecifics. Probiotic-enhanced immune tolerance (inhibition of host-versus-graft) via IL-10 permits eutherian mammals to sustain a prolonged placental pregnancy. Hyperacidic mucus inhibits pathogens that otherwise impede GI tract health, fertilization and pregnancy. Under favorable conditions, these probiotic bacteria are then passed from mother to naïve offspring during vaginal birth and nursing, imparting evolutionary success to both the symbiotic bacteria and their mammalian hosts.

### Experimental Procedures

#### Animals

C57BL/6 wild type (wt) and IL10-deficient mice (Jackson labs; Bar Harbor, ME), and outbred CD-1 (Charles River; Wilmington, MA) mice were housed and handled in Association for Assessment and Accreditation of Laboratory Animal Care (AAALAC)-accredited facilities using techniques and diets enriched with probiotic yogurt or *Lactobacillus reuteri* as pre-approved by Massachusetts Institute of Technology’s Committee on Animal Care (CAC). Mice were bred in-house to achieve experimental groups. Each experiment included 5–10 animals per group with two replications (total N = 10–20 mice per group). Mice were fed standard rodent chow (MRH 3000; Purina Labs, St Louis MO) unless otherwise specified. Tissues for analyses were collected upon necropsy.

#### Human subjects and specimen collection

Human subject measurements of vaginal pH, *Lactobacillus sp* abundance, and age were obtained from Ravel *et. al*., 2011 [12]. To control for the known effects of ethnicity on vaginal pH, we used only the 97 caucasian subjects enrolled in this study, which represented the dominant ethnic group. Statistical differences between groups were assessed using the Mann-Whitney U test.

#### Special diets for animals

CD-1 mice of age = 6–8 wks were receiving an alternative control diet AIN-76A (Harlan-Teklad Madison WI) for 90 days prior to microscopic examination of skin at age = 20 wks.

#### Probiotic treatments

Subgroups were supplemented with a commercially available vanilla-flavored probiotic yogurt (0.8 ml/mouse 3X weekly). Separate groups received an anti-inflammatory strain of *Lactobacillus reuteri* ATCC 6475 cultivated as described elsewhere [23] using a dosage of 3.5×10^5^ organisms/mouse/day in drinking water.

#### Scoring of fur luster

Relative fur luster, or ‘shininess’ of the fur was quantified using a combination of mechanical and sensory methods. Mechanical evaluation was under fixed illumination using a Gossen Starlight II Model 4046 lightmeter (Gossen; Nurenberg, Germany), an optical instrument used in precision photography and photometry to accurately measure reflected light. Using the viewfinder, digital measurement were taken with either a 1 degree and 5 degree field of view. Surrounding visual fields were blocked with a matte black substrate for uniformity. In addition, under fixed illumination a team of human panelists who were blinded to sample identity provided sensory scores of hair luster, similar to sensory systems used by cosmetics companies to evaluate glossy hair [46], based upon a luster rating scale of 1 = shiniest to 5 = dullest that was reproducible among panelists.

#### Evaluation of pH levels in mice

In order to test the pH of mouse mucous membranes or skin, a pH electrode (Orion model 320 meter) was calibrated per manufacturer’s instructions at the outset of every assay. Distilled water for sample immersion was also calibrated to standard pH = 7.00 at the outset of every assay. Four tissue sites: oral cavity, skin behind the ear, the vaginal cavity (females) or inner prepuce (males), and rectum from each mouse were collected with cotton-tipped swabs and immersed in 0.4 ml of dH2O using 24 well plates. The pH electrode was rinsed thoroughly with distilled water between readings. Resulting pH values were recorded digitally for each body site, and then compared between treatment groups.

#### Systemic depletion of interleukin-17A using anti-cytokine antibodies

Mice at age 6 months or older were treated with intraperitoneal injection of anti-IL17A antibody (clone 17F3; BioXcel, Lebanon, NH) at 500 µg per mouse three times weekly for three-four weeks. Mice were then euthanized and compared with age-matched mice that received isotype-matched sham IgG antibody alone.

#### Histopathology and immunohistochemistry

For histologic evaluation, four sequential oblong skin samples, collected from the same area of the dorsolateral abdominal region of each mouse upon necropsy, were fixed in formalin. Samples from each mouse were embedded together in paraffin and cut at 5 µm. Serial sections were stained with hematoxylin and eosin and immunohistochemistry (IHC). Rabbit monoclonal anti-Ki-67 (Cell Marque, Rocklin, CA) or polyclonal anti-cleaved caspase-3 (Cell Signaling, Beverly, MA) antibodies were used for IHC. Heat-induced antigen retrieval was performed with CC1 epitope retrieval solution (Ventana Medical Systems, Inc., Tucson, AZ) for ki-67 or with citrate buffer pH6 for caspase-3.

Quantitative histomorphometry was done in twenty microscopic fields/images captured per experimental group for each assessment using the Image J image processing and analysis program (NIH, Bethesda, MD). Subcutaneous hair follicle profiles and skin thickness were quantified in low (×4) whereas total sebocytes and ki-67+ sebocytes in high (×20) magnification images.

Hair follicle staging was performed in fifty longitudinally-oriented hair follicles per experimental group. The cycle stage of each hair follicle was recognized based on the combination of histomorphological criteria together with its proliferation and apoptosis immunohistochemical profile [19].

### Statistical Analyses

Data from mouse models were evaluated for normality with Shapiro-Wilk test. Subcuticular hair follicles**,** skin thickness and sebocyte counts were analyzed with the Mann Whitney U test for non-parametrical data. pH measurements and reflectometry data analysis was done with one way analysis of variance (ANOVA) and Tukey’s multiple comparison post test. Hair follicle staging comparison between groups was done with the Chi-square test. All the above mentioned analyses were performed with the Graphpad Prism version 5.0 for Windows, GraphPad software, San Diego, CA, USA. Fur luster panelists’ scoring data were analyzed with the Generalized Linear Mixed Models (GLMMs) procedure with animals considered as a random factor and treatment and scores as fixed, using GENSTAT software (Version 5.0, Lawes Agricultural Trust, Rothamstead, U.K.). The logarithm was used as a link function [47]. The GLMMs analysis was followed by a t-test to calculate pairwise differences between means. Effects were considered to be significant at p<0.05.
